# Campylobacter rectus Thoracic Empyema in a Severely Malnourished Host With Preserved Conventional Immune Markers: A Case Report and Literature Review

**DOI:** 10.7759/cureus.116841

**Published:** 2026-09-24

**Authors:** Janiza Anne Claire M Marcelo, Gabriela Carrillo, Raul Magadia

**Affiliations:** 1 Internal Medicine, University of Santo Tomas, Manila, PHL; 2 Medicine, Alabama College of Osteopathic Medicine, Dothan, USA; 3 Infectious Disease, Regional Medical Center, Anniston, USA

**Keywords:** anaerobic pathogens, bacterial empyema, campylobacter rectus, immunocompetent host, loculated pleural effusion, malnutrition risk, oral-type pleural infection, video-assisted thoracoscopic surgery (vats)

## Abstract

*Campylobacter rectus* is a fastidious, anaerobic to microaerophilic gram-negative bacillus best recognized as a key periodontopathogen residing in the oral cavity. This organism is generally confined to the subgingival flora and rarely causes disease beyond the oral cavity. However, invasive extraoral infection with *C. rectus *has been described in isolated case reports, and thoracic empyema caused by this organism remains exceptionally rare, with diagnosis often delayed due to its fastidious growth requirements and low clinical suspicion. We present the case of a malnourished patient with poor dentition who developed thoracic empyema due to *C. rectus*. This case underscores the importance of considering fastidious oral commensals in the differential diagnosis of culture-negative empyema and highlights poor dentition as an under-recognized source of invasive infection in vulnerable hosts.

## Introduction

Pleural infection continues as a clinically important cause of morbidity and mortality. Empyema, defined by the presence of pus or bacteria within the pleural space, usually develops as a complication of pneumonia and progresses through exudative, fibrinopurulent, and organizing stages [1-3]. Delay in source control may lead to loculation, visceral pleural peel formation, impaired lung re-expansion, and the need for surgical drainage [1,3].

The microbiology of pleural infection varies according to community-acquired versus hospital-acquired setting. Community-acquired disease is commonly associated with streptococci and staphylococci, but anaerobes are increasingly recognized, particularly in aspiration-related cases and in patients with poor oral hygiene [2-4]. These infections may present more indolently than typical pyogenic infections, with less fever and more constitutional weight loss [4].

*Campylobacter rectus* occupies a specialized niche within this system. Unlike *Campylobacter jejuni *and *Campylobacter coli*, which are primarily associated with foodborne gastroenteritis, *C. rectus *is principally an oral organism associated with gingivitis and periodontitis [5,6]. Extraoral invasive disease is rare. Reported thoracic infections due to *C. rectus *suggest an oral-to-pulmonary route, usually through microaspiration of colonized oropharyngeal secretions rather than hematogenous dissemination from the gastrointestinal tract [7-11].

We describe a case of monomicrobial *C. rectus *thoracic empyema in a severely malnourished man with suspected metastatic malignancy and preserved conventional immune markers. The case is notable for its microbiologic rarity, the absence of bacteremia or enteric *Campylobacter *isolation, the requirement for video-assisted thoracoscopic surgery (VATS) decortication, and the more general question of whether severe malnutrition should be considered a form of functional immunocompromise even when standard immunologic tests are normal.

## Case presentation

A 60-year-old man with no established prior medical history presented in early March 2026 with progressive dyspnea and left-sided pleuritic chest pain. He reported approximately three months of unintentional weight loss, chronic loose stools, and declining functional capacity. He denied fever at home, hemoptysis, sick contacts, recent dental procedures, previous hospitalization, corticosteroid exposure, chemotherapy, or other immunosuppressive therapy.

On examination, he was markedly cachectic and had peripheral anasarca. His body mass index was 14 kg/m², consistent with severe protein-calorie malnutrition. Oral examination demonstrated advanced carious dentition without a gross periodontal abscess. Breath sounds were markedly decreased at the left lung base with dullness to percussion.

Initial laboratory evaluation showed a white blood cell count of 5.8×10³/µL, which was unexpectedly normal given the severity of pleural infection and may have reflected impaired inflammatory reserve in the setting of severe malnutrition. Serum creatinine was 0.59 mg/dL, and transaminases and bilirubin were within normal limits.

Chest radiography demonstrated a large left pleural effusion with associated left lower lobe atelectasis (Figure 1). Computed tomography (CT) angiography of the chest showed no pulmonary embolism but confirmed a large loculated left pleural effusion with compressive collapse of the left lower lobe (Figure 2). A small right pleural effusion and diffuse emphysematous changes were also present (Figure 3). CT imaging of the abdomen showed diffuse heterogeneous increased osseous density, raising concern for skeletal metastatic disease (Figure 4). Prostate-specific antigen was subsequently elevated, and outpatient prostate biopsy was planned.

**Figure 1:**
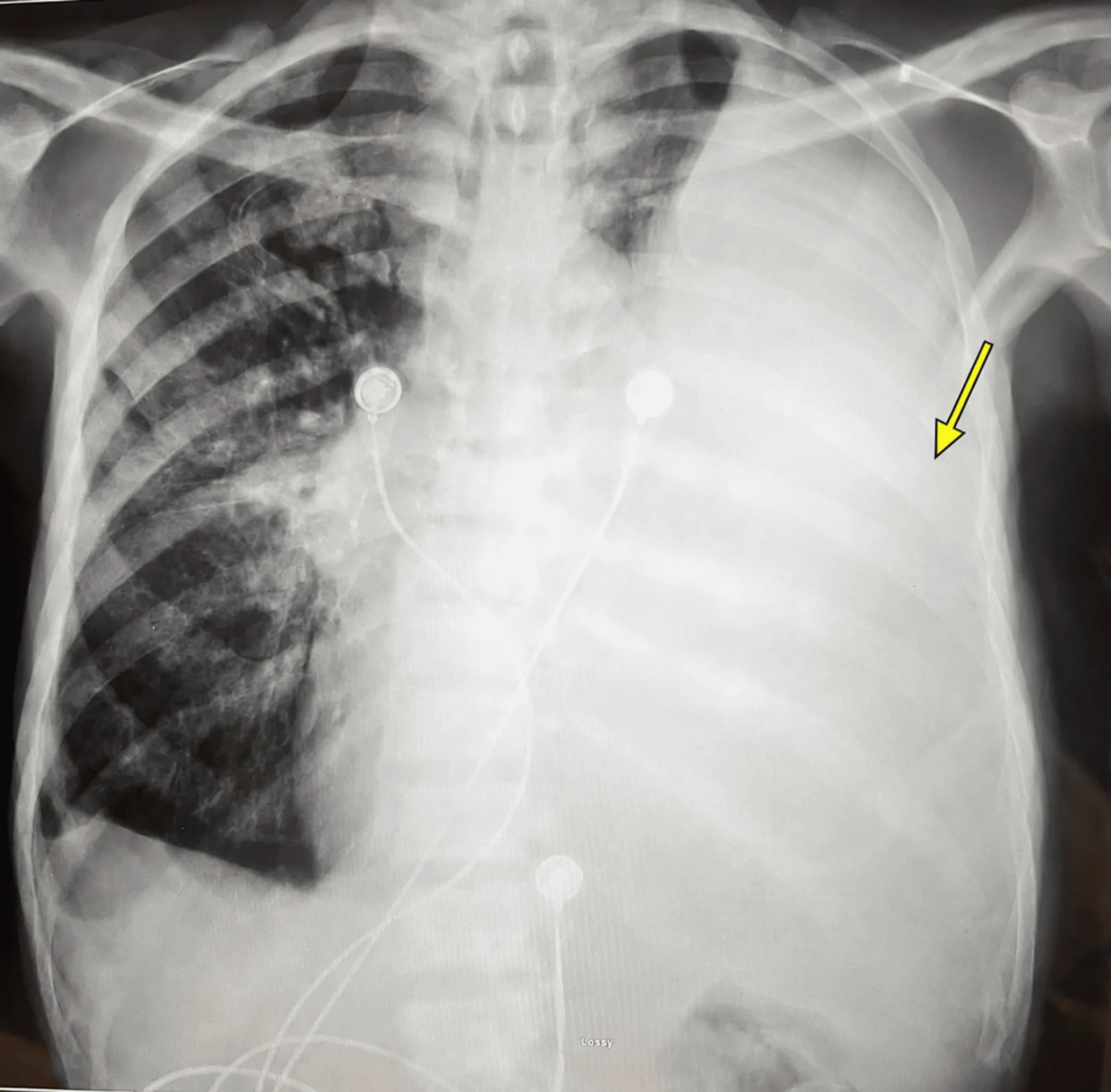
Initial chest radiograph demonstrating a large left pleural effusion with near-complete opacification of the left hemithorax (yellow arrow) and associated left lung atelectatic changes

**Figure 2:**
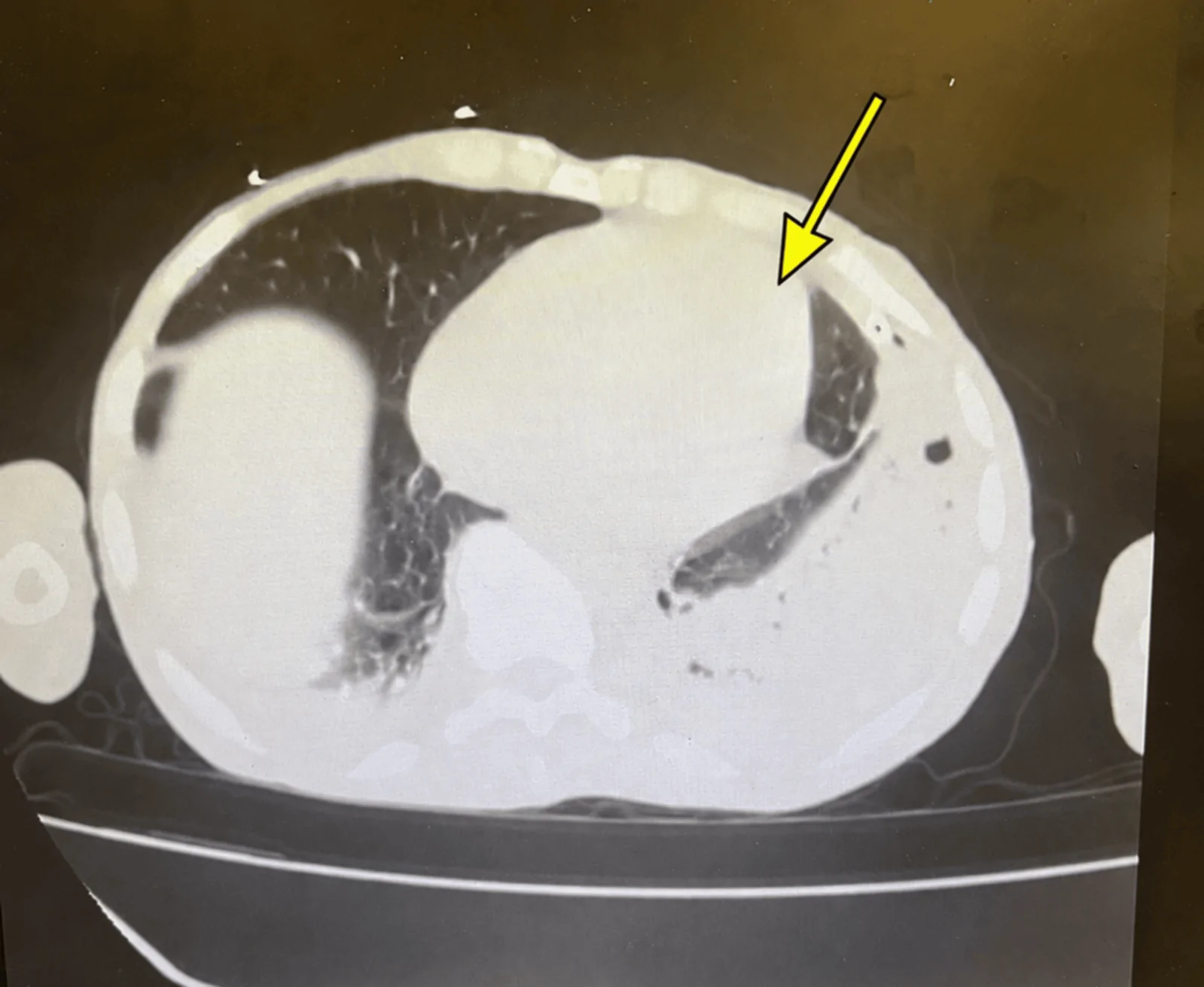
Computed tomography of the chest demonstrating a large left pleural effusion (yellow arrow) with associated compressive atelectatic changes of the left lower lung

**Figure 3:**
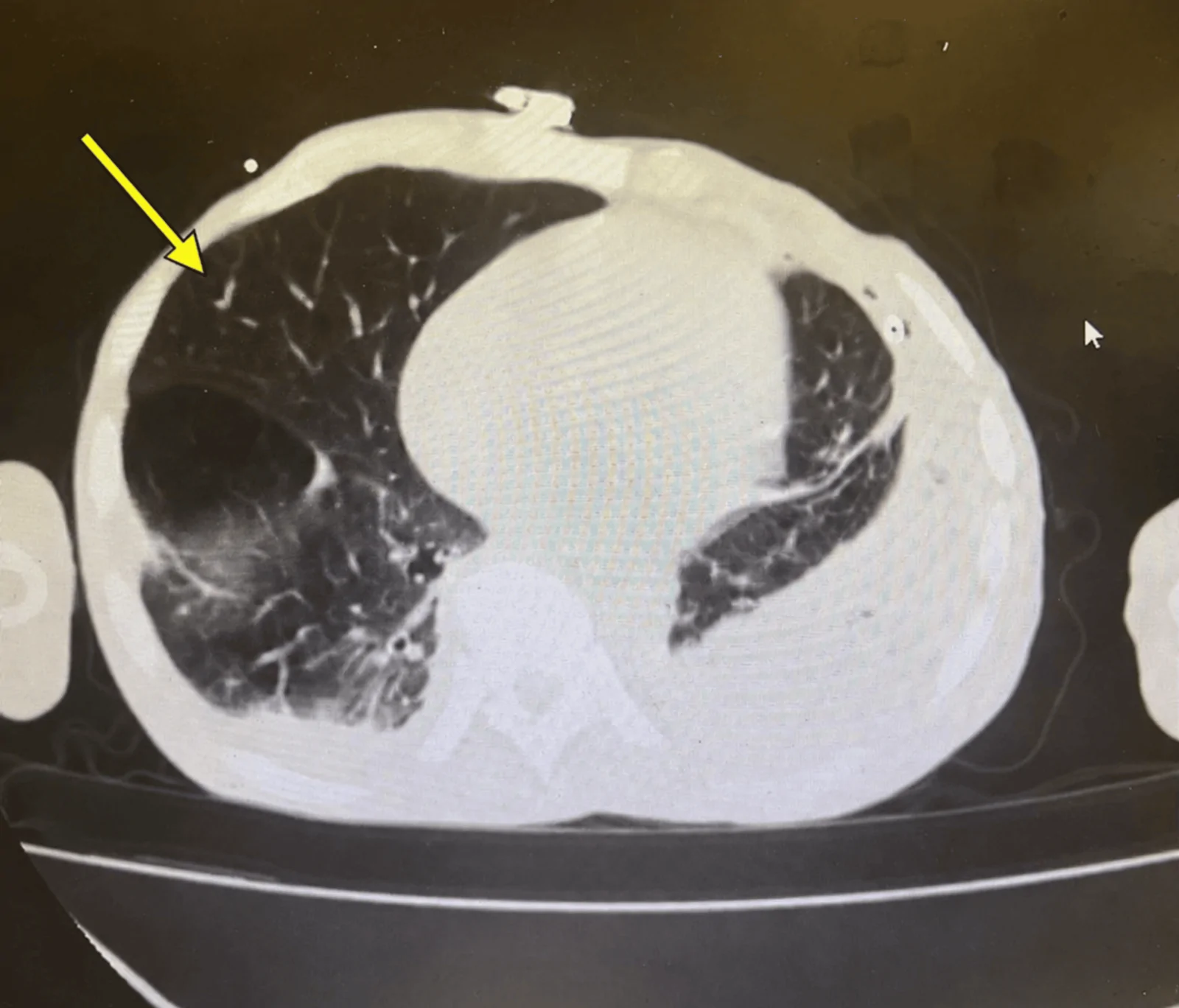
Computed tomography of the chest demonstrating diffuse emphysematous changes (yellow arrow)

**Figure 4:**
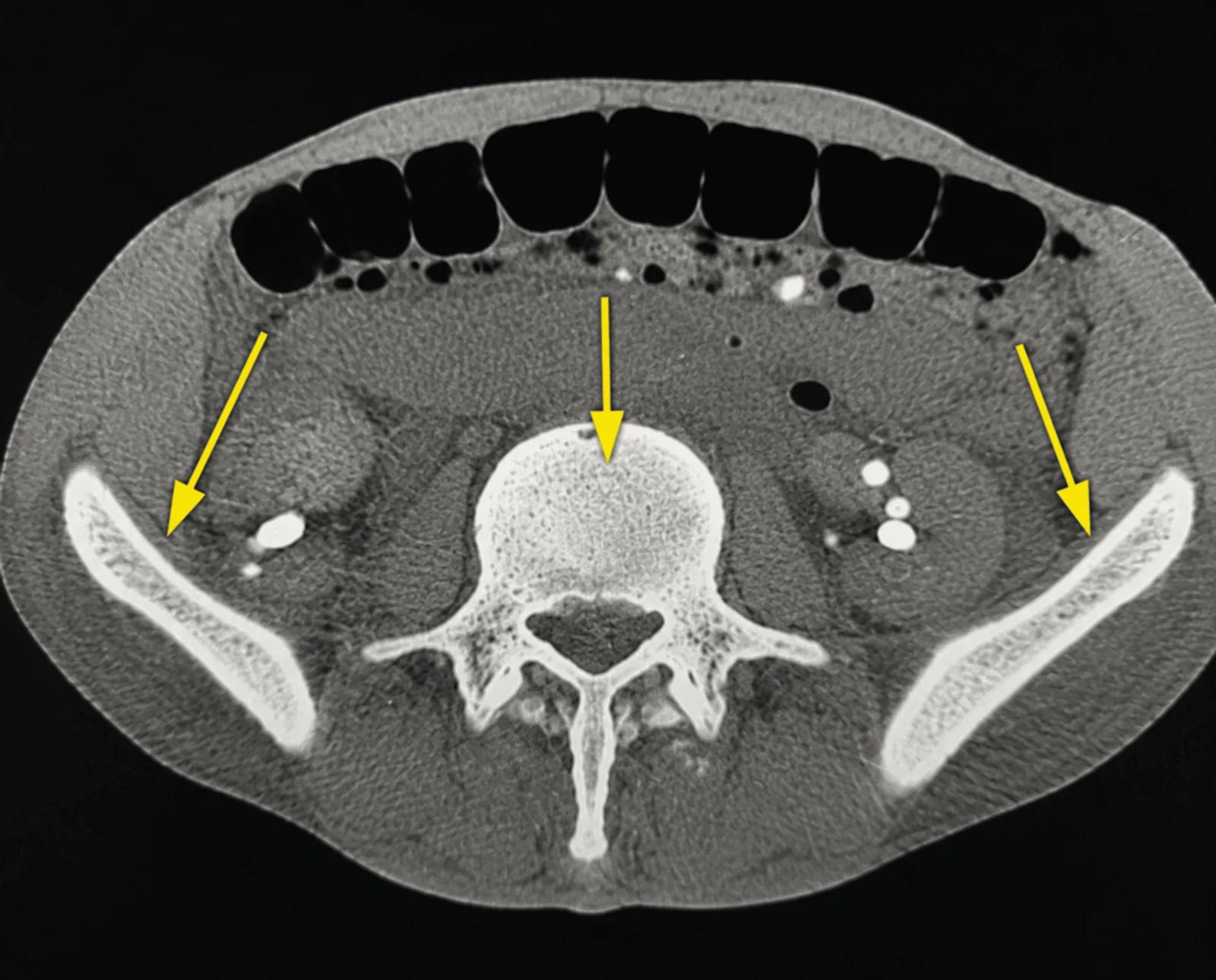
Computed tomography of the abdomen demonstrating diffuse heterogeneous increased osseous density (yellow arrows)

Two sets of peripheral blood cultures were obtained and remained negative. On hospital day 2, thoracentesis yielded frankly purulent pleural fluid, and a left-sided chest tube was placed. Pleural fluid analysis confirmed empyema, with a white blood cell count of 90,694/µL, 91% neutrophils, red blood cell count of 21,000/µL, lactate dehydrogenase of 4,500 IU/L, and glucose <4 mg/dL (Table 1).

**Table 1:** Pleural fluid analysis Pleural fluid demonstrated a markedly inflammatory, exudative profile consistent with complicated parapneumonic effusion/empyema, with *Campylobacter rectus *identified as the sole isolate. Concurrent blood and stool cultures were negative.

Parameter	Result	Reference range/diagnostic threshold	Interpretation
Appearance	Frankly purulent, turbid, yellow	Clear, pale yellow	Consistent with established empyema
White blood cell count	90,694/µL	<1,000/µL	Marked neutrophilic pleocytosis
Differential	91% neutrophils, 9% lymphocytes	Neutrophils <50%	Acute bacterial pleural infection
Red blood cell count	21,000/µL	<5,000-10,000 (non-hemorrhagic)	Hemorrhagic component, nonspecific
Lactate dehydrogenase	4,500 IU/L	Pleural fluid/serum lactate dehydrogenase ratio >0.6, or pleural lactate dehydrogenase >2/3 upper limit of normal serum lactate dehydrogenase, defines an exudate (Light's criteria); >1,000 IU/L supports a complicated effusion	Markedly elevated
Glucose	<4 mg/dL	Approximates serum glucose (60-100 mg/dL); <40-60 mg/dL defines a complicated parapneumonic effusion/empyema	Advanced complicated parapneumonic effusion/empyema
Culture	Campylobacter rectus	No growth (sterile)	Sole isolate
Blood culture	Negative ×2 sets	No growth	No bacteremia documented
Stool culture	Negative, including *Campylobacter *	No growth	Supports a non-enteric source

Pleural fluid culture, after extended incubation under appropriate anaerobic/microaerophilic conditions, yielded *C. rectus *as the sole isolate. No co-pathogens were identified. Blood cultures remained negative throughout hospitalization. Stool cultures, including targeted *Campylobacter *culture, were negative, arguing against enteric campylobacteriosis or hematogenous spread from a gastrointestinal reservoir.

Empiric broad-spectrum therapy was initially given with piperacillin-tazobactam. After speciation, treatment was narrowed to intravenous ampicillin-sulbactam with oral azithromycin, guided by the organism's reported susceptibility patterns and published experience with invasive *C. rectus *infection.

An in-depth evaluation for immunodeficiency was performed. HIV antigen/antibody testing, rapid plasma reagin, and hepatitis B and C serologies were negative. Serum immunoglobulin levels, IgG subclasses, and CD4/CD8 lymphocyte counts were within reference ranges.

Despite chest tube placement, drainage remained inadequate because of loculated, organized pleural infection (Figure 5). On hospital day 12, the patient underwent VATS with decortication. The chest tube was removed on hospital day 19. He demonstrated clinical and biochemical improvement and was tolerating ampicillin-sulbactam plus azithromycin on day 13 of a planned 21-day antimicrobial course at the time of report preparation (Figure 6).

**Figure 5:**
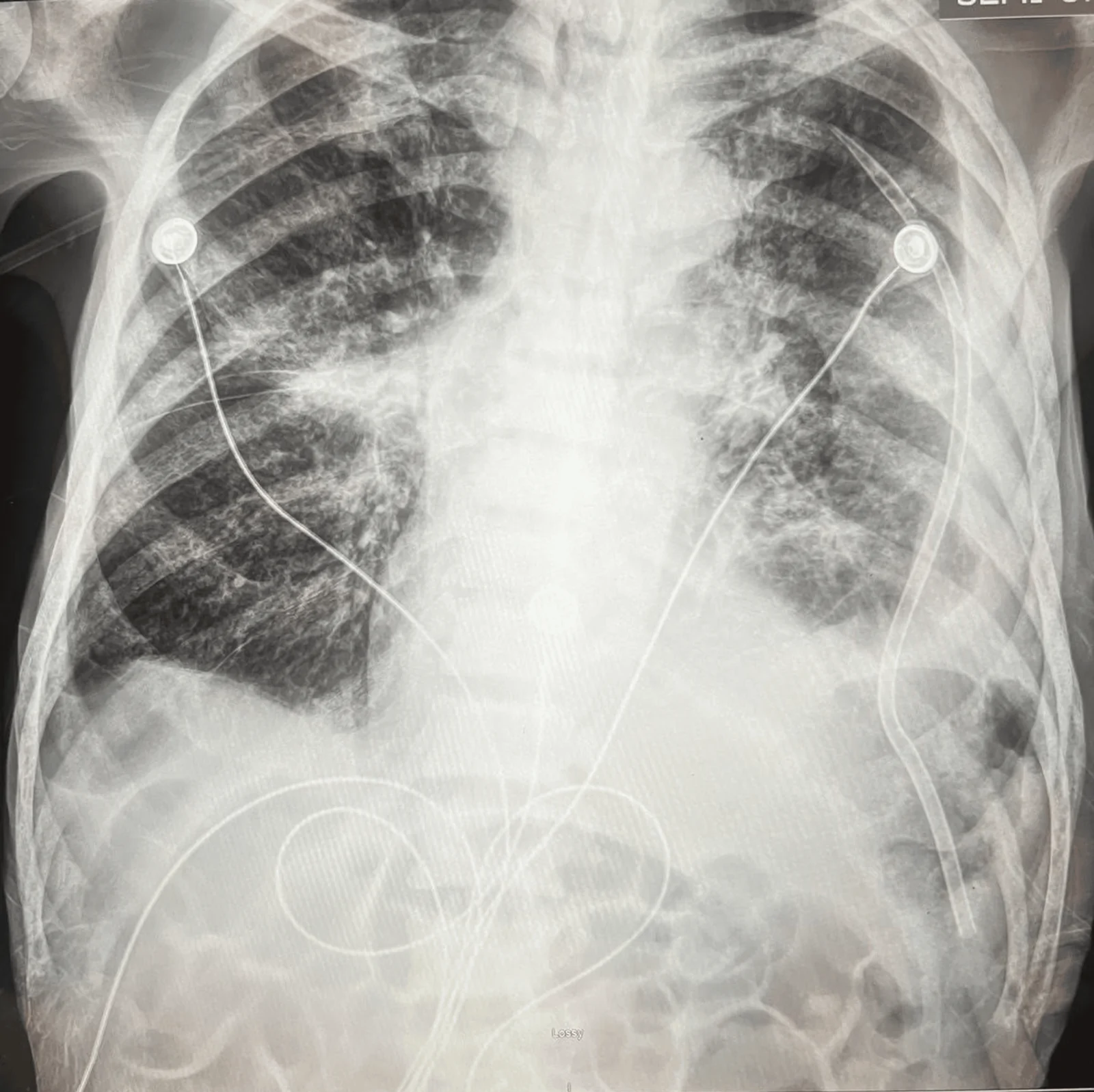
Post-procedural chest radiograph demonstrating the interval placement of a left thoracostomy tube with persistent left basilar pleural fluid, consistent with residual pleural effusion

**Figure 6:**
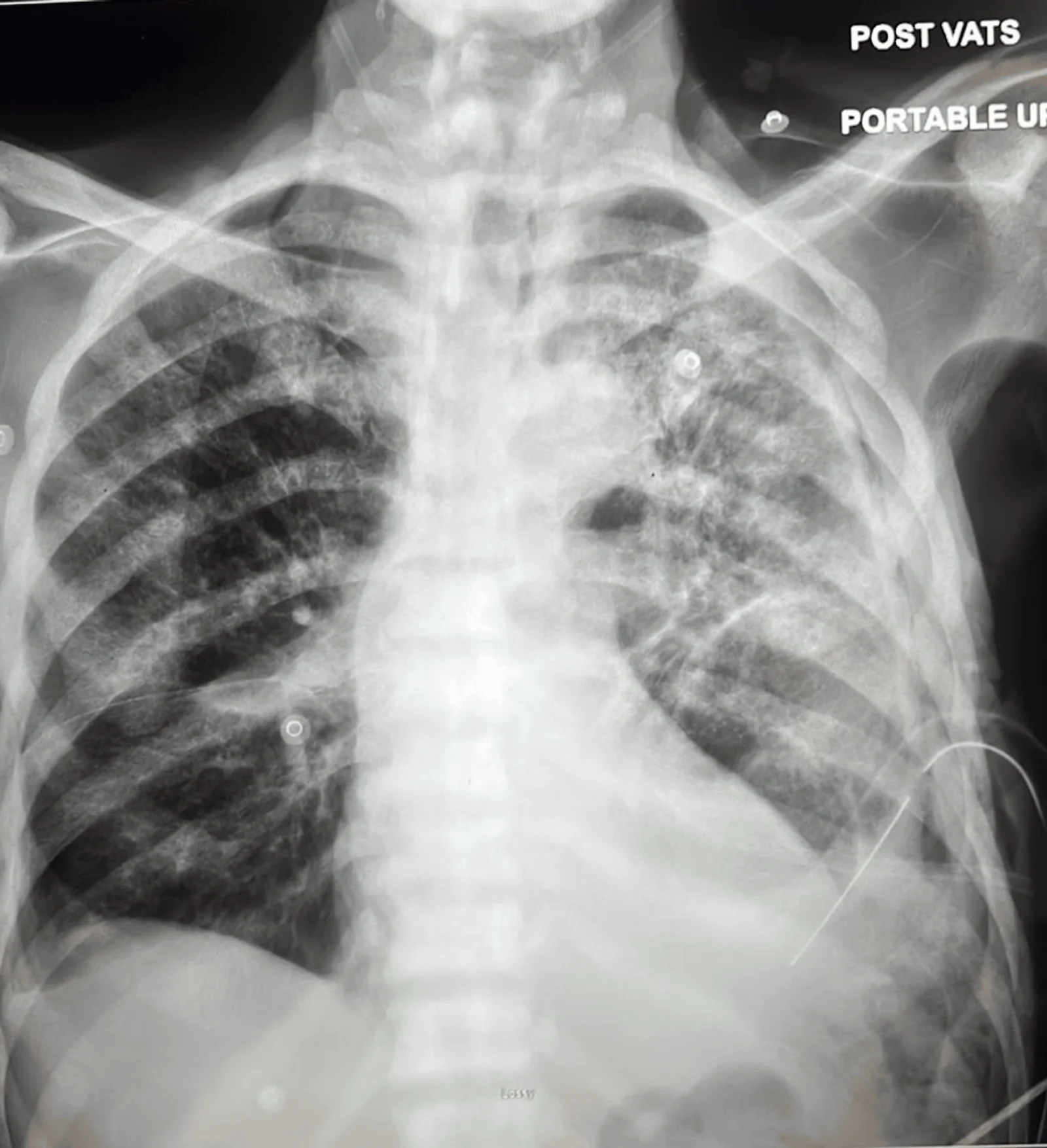
Portable chest radiograph following VATS demonstrating decreased left pleural effusion with a left chest tube in place VATS: video-assisted thoracoscopic surgery

## Discussion

This case describes a 60-year-old man with profound malnutrition, poor dentition, and suspected occult malignancy who developed severe loculated pleural empyema due to *C. rectus*, successfully treated with surgical source control and antimicrobial therapy. This case illustrates four clinically relevant themes: the invasive potential of oral anaerobic flora, the diagnostic difficulty posed by fastidious organisms, the value of prompt source control in loculated pleural infection, and the need to recognize severe malnutrition as a meaningful form of host vulnerability even when conventional immune markers are preserved.

*C. rectus *is a fastidious, gram-negative, anaerobic to microaerophilic bacillus that is primarily recognized as an oral commensal and periodontopathogen associated with periodontal disease [4-6,8,10,11]. Although *C. rectus *has increasingly been implicated in extraoral infections, invasive disease remains uncommon, and pleural space infection is particularly rare. Published reports of *C. rectus *empyema remain limited, and most cases have involved older adults, poor dentition, aspiration risk, or co-isolation of other oral anaerobes [7-12]. This case highlights an unusual presentation of *C. rectus *as the sole pathogen responsible for a severe thoracic empyema and underscores the importance of considering oral flora in patients with pleural infection, particularly when predisposing factors for microaspiration are present. 

The negative blood cultures and negative stool cultures support direct oral-to-pulmonary translocation rather than hematogenous dissemination from the gastrointestinal tract. This distinction is important because the patient's chronic diarrhea could initially suggest enteric *Campylobacter *infection. However, *C. rectus *is biologically and epidemiologically distinct from common enteric *Campylobacter *species [5]. In this case, chronic diarrhea and weight loss were more plausibly related to severe systemic illness, malnutrition, and suspected underlying malignancy.

An additional feature of this case is the patient's profound protein-calorie malnutrition, with a body mass index of 14 kg/m², substantial unintentional weight loss, and concern for occult malignancy. Although formal immunologic evaluation, including HIV testing, quantitative immunoglobulins and subclasses, and CD4/CD8 lymphocyte counts, did not demonstrate a conventional immunodeficiency, severe malnutrition may impair host defense through mechanisms that are not captured by routine immune testing [13,14]. Malnutrition is capable of impairing epithelial and mucosal barriers, cellular immunity, antibody responses, complement activity, cytokine signaling, and phagocyte function [13,14].

The suspected metastatic malignancy further increases this vulnerability. Advanced cancer can produce immune dysregulation, cachexia, and impaired innate immune monitoring before chemotherapy or corticosteroids are introduced [14]. This case therefore supports describing the patient as "without a discrete laboratory-defined immunodeficiency" rather than simply "immunocompetent". 

The antimicrobial management of *C. rectus *infections is not well standardized because invasive infections are rare and published experience is limited. Published case experience and in vitro susceptibility reports generally support activity of penicillins, beta-lactam/beta-lactamase inhibitor combinations, carbapenems, clindamycin, and macrolides, while fluoroquinolone susceptibility may be variable across *Campylobacter *species [7-11,15,16]. Treatment should therefore be guided by the site and severity of infection, susceptibility data when available, and, most importantly, adequate source control. In this case, the patient was empirically initiated on piperacillin-tazobactam given the severity of the pleural infection and the initially uncertain microbiologic etiology, with coverage for anaerobic and other potential respiratory pathogens. Following the identification of *C. rectus*, antimicrobial therapy was de-escalated to ampicillin-sulbactam with oral azithromycin, reflecting targeted antimicrobial stewardship based on the identified pathogen. Azithromycin added overlapping anti-*Campylobacter *activity, consistent with reported approaches to invasive *Campylobacter *infections [7-11,15,16]. However, given inadequate drainage with initial chest tube placement and the presence of a large, loculated empyema, definitive source control required VATS with decortication. The patient's subsequent clinical improvement reinforces that antimicrobial therapy alone is insufficient in organized empyema; durable recovery is dependent on both active antimicrobial therapy and mechanical source control [1-3].

## Conclusions

We report a rare case of monomicrobial *C. rectus *thoracic empyema in a severely malnourished man with poor dentition, suspected metastatic malignancy, and preserved conventional immune markers. The case supports an oral-pleural pathogenesis through microaspiration and draws attention to the need to consider oral anaerobes in cryptogenic or aspiration-associated empyema. It also argues for a more detailed view of host susceptibility: severe malnutrition and cancer-associated cachexia may represent clinically meaningful functional immunocompromise even when standard immunologic testing is normal. Successful management required specialized microbiologic recovery, targeted antimicrobial therapy, and definitive source control with VATS decortication.

## References

[1] Shen KR, Bribriesco A, Crabtree T (2017). The American Association for Thoracic Surgery consensus guidelines for the management of empyema. J Thorac Cardiovasc Surg.

[2] Davies HE, Davies RJ, Davies CW (2010). Management of pleural infection in adults: British Thoracic Society Pleural Disease Guideline 2010. Thorax.

[3] Roberts ME, Rahman NM, Maskell NA (2023). British Thoracic Society guideline for pleural disease. Thorax.

[4] Bartlett JG (1993). Anaerobic bacterial infections of the lung and pleural space. Clin Infect Dis.

[5] Macuch PJ, Tanner AC (2000). Campylobacter species in health, gingivitis, and periodontitis. J Dent Res.

[6] Cortelli JR, Aquino DR, Cortelli SC (2008). Etiological analysis of initial colonization of periodontal pathogens in oral cavity. J Clin Microbiol.

[7] Lam JY, Wu AK, Ngai DC (2011). Three cases of severe invasive infections caused by Campylobacter rectus and first report of fatal C. rectus infection. J Clin Microbiol.

[8] Ogata T, Urata T, Nemoto D, Hitomi S (2017). Thoracic empyema caused by Campylobacter rectus. J Infect Chemother.

[9] Noël A, Verroken A, Belkhir L, Rodriguez-Villalobos H (2018). Fatal thoracic empyema involving Campylobacter rectus: a case report. Anaerobe.

[10] Matsumoto R, Himeji D, Shiiba R (2022). Thoracic empyema caused by Campylobacter rectus. Intern Med.

[11] Figueiredo L, Ferrão J, Ferreira C, Fernandes A, Costa MJ (2022). A rare case of pleural empyema caused by Campylobacter rectus. Cureus.

[12] Gray RM, Vidwans M (2019). Mixed anaerobic thoracic empyema: the first report of Filifactor alocis causing extra-oral disease. New Microbes New Infect.

[13] Keusch GT (2003). The history of nutrition: malnutrition, infection and immunity. J Nutr.

[14] Saunders J, Smith T (2010). Malnutrition: causes and consequences. Clin Med (Lond).

[15] Horio Y, Shiraishi Y, Watanabe N, Inoue S, Imanishi T, Asano K (2017). Empyema associated with Campylobacter curvus infection. Respirol Case Rep.

[16] Nagao K, Shimoda M, Ito M, Shimoda K, Morimoto K, Yoshimori K, Tanaka Y (2026). Campylobacter coli empyema resistant to macrolides and fluoroquinolones: a case report and literature review. Intern Med.

